# Compensatory Growth Induced in Zebrafish Larvae after Pre-Exposure to a *Microcystis aeruginosa* Natural Bloom Extract Containing Microcystins

**DOI:** 10.3390/ijms10010133

**Published:** 2009-01-05

**Authors:** Issam El Ghazali, Sanaa Saqrane, Antonio Paulo Carvalho, Youness Ouahid, Brahim Oudra, Francisca Fernandez Del Campo, Vitor Vasconcelos

**Affiliations:** 1 Department of Biology, Laboratory of Biology and Biotechnology of Microorganisms, Microbiology and Environmental Toxicology Unit, Faculty of Sciences Semlalia Marrakech, University Cadi Ayyad, P.O. Box 2390, Marrakech 40000, Morocco. E-Mails: elghazali@gmail.com (I. E-G.); saqranesanaa@yahoo.fr (S. S.); oudas02@yahoo.fr (B. O.); 2 Centro Interdisciplinar de Investigação Marinha e Ambiental, CIIMAR/CIMAR-LA, Rua dos Bragas 289, Porto 4050-123, Portugal. E-Mail: apcarval@fc.up.pt (A. P. C.); 3 Departamento de Zoologia e Antropologia, Faculdade de Ciências, Universidade do Porto, Rua do Campo Alegre, 4169-007 Porto, Portugal; 4 Departamento de Biologia, Laboratorio de Fisiologia Vegetal, Universidad Autonoma de Madrid, Cantoblanco, 28049 Madrid, Spain. E-Mails: youness.ouahid@uam.es (Y. O.); pacuchi@gmail.com (F. F-C.)

**Keywords:** Cyanobacterial bloom extract, Natural risk, Microcystins, Malformations, Compensatory growth

## Abstract

Early life stage tests with zebrafish (*Danio rerio*) were used to detect toxic effects of compounds from a *Microcystis aeruginosa* natural bloom extract on their embryolarval development. We carried out the exposure of developing stages of fish to complex cyanobacterial blooms containing hepatotoxic molecules - microcystins. Fish embryo tests performed with the bloom extract containing 3 mg·L^−1^ Eq microcystin-LR showed that after 24 h of exposure all fish embryos died. The same tests performed with other diluted extracts (containing 0.3, 0.1 and 0.03 mg·L^−1^ Eq microcystin-LR) were shown to have an influence on zebrafish development and a large number of embryos showed malformation signs (edema, bent and curving tail). After hatching the larvae were transferred to a medium without toxins to follow the larval development under the new conditions. The specific growth of the pre-exposed larvae was significantly more important than that of the control larvae. This may represent a compensatory growth used to reduce the difference in size with the control fish noted after hatching.

## 1. Introduction

The increase of population and the consequent intensification of agricultural and industrial activities have led to the enhancement of eutrophication in superficial freshwater bodies, and then it has led to more frequent worldwide cyanobacteria blooms, producing a variety of toxins, including hepatotoxins, neurotoxins and dermatotoxins [1]. Among hepatotoxins, microcystins are the most widely distributed [2], being produced mainly by freshwater cyanobacteria such as *Microcystis*, *Oscillatoria*, *Nostoc*, *Aphanizomenon* and *Anabaena* [3, 4]. Over 70 different structural analogues of microcystins have been isolated from natural blooms or laboratory cultures of cyanobacteria [5, 6, 7].

Microcystins produce toxic effects in both animals and humans, being actually associated with fish kills [8, 9]. Microcystins affect in fish a large number of organs, such as liver, intestine, kidney [10], heart [11], gills [8, 12]. Haematological disorders [13] and serum enzymes elevations [8] have also been observed. Several hepatobiliary abnormalities such as liver hypertrophy and hepatic haemorrhage were recently described in fish embryos microinjected with microcystins [14]. However, freshwater fish as well as other aquatic animals, could not only be damaged by cyanobacterial toxins but are also able to bioaccumulate them [15], representing a human health risk the ingestion of contaminated food. So far little information is available on the effects of cyanobacterial toxins on the embryonic development of aquatic animals [16–19]. Indeed, these toxicity studies have given little evidence of whether microcystins have adverse effects on development of aquatic species. The embryos may be more susceptible to cyanobacterial toxins than juvenile and adult fish, as described for many xenobiotics [20].

In aquatic environments, surface aggregations of some cyanobacteria may accumulate as scum with high cell densities and toxin concentrations. This phenomenon often occurs in shallow littoral areas, which are the primary environments for the early life stages of aquatic vertebrates. Such extreme conditions can lead to significant exposures and various chronic effects or event deaths of aquatic organisms [5, 21]. Early-life stage development plays a crucial role in the ontogenesis of aquatic organisms and the embryolarval tests that model these steps of development were successfully used to study cyanobacterial toxicity in fish.

Previous reports have shown minor toxic effects of purified microcystins and aqueous cyanobacterial extracts on embryolarval development of zebrafish (*Danio rerio*), rainbow trout (*Oncorhynchus mykiss*) or chub (*Leuciscus cephalus*) [16, 17]. On the other hand, significant toxicities were shown for mud loach *Misgurnus mizolepis* [22]. It has also been shown that fish eggs are well protected against the toxic effects of pure microcystins, only microinjection applications of microcystin-LR caused mortality in zebrafish [23] and medaka (*Oryzias latipes*) [14]. Some researches indicate that cyanobacterial substances other than microcystins may have more significant effects on the development of *Daphnia* and *Artemia* [24, 25, 26].

Since crude cyanobacterial extracts may be released into water during the decay of cyanobacterial blooms and may possibly contain toxic compounds in addition to the known toxins, the aim of this study was to investigate the direct effects of a natural *Microcystis aeruginosa* bloom extract on zebrafish (*Danio rerio*) embryos and to follow the growth of the newly hatched larvae without exposure to the bloom material.

## 2. Results and Discussion

**Figure 1.**
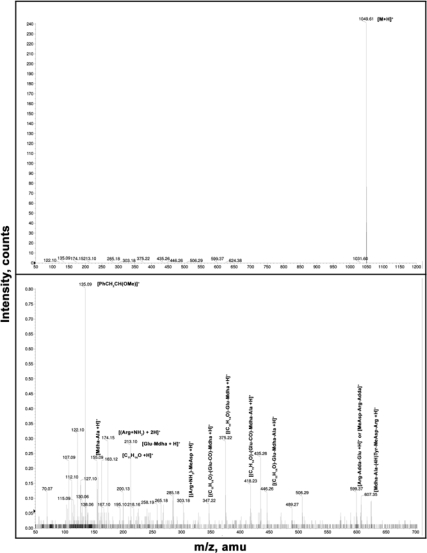
Full scan mass spectra of MC(H4)-YR.

The lyophilized *Microcystis aeruginosa* bloom from Lalla Takerkoust reservoir had a total toxin content of 976 μg Eq MC-LR.g^−1^ dry weight. Five microcystins variants (MC-RR; MC-YR; MC-LR; MC-FR; MC-WR) were detected in the sample by HPLC-PDA analysis using microcystin standards. The sixth and last microcystin variant (MC-(H4)YR) was identified by Liquid Chromatography-Mass Spectrometry (LC-MS) analysis (Figure 1).

**Figure 2.**
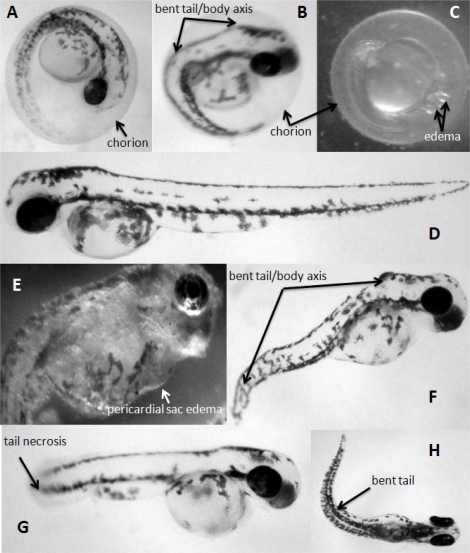
Effects of *Microcystis aeruginosa* natural bloom extract on the embryonic and larval development of the zebrafish (*Danio rerio*): **A**: control (56 h); **B**: treated embryo (56 h); **C**: treated embryo (56 h); **D**: control (84 h); **E**: pre-exposed larvae (146 h); **F**: pre-exposed larvae (98 h); **G**: pre-exposed larvae (84 h); **H**: pre-exposed larvae (98 h); arrows mark different types of malformations.

The exposure of zebrafish eggs to the bloom extracts increased the time of beginning of hatching in all treatments and this effect was significantly correlated with the microcystin content of the bloom extracts used in this test (Pearson’s r = 0.984, P < 0.05). The time necessary to complete eggs hatching also increased (Table 1). The exposure to *Microcystis aeruginosa* natural bloom extract containing 3 mg·L^−1^ Eq MC-LR prevented the hatching of all eggs, and in all other treatments the hatching rate was reduced significantly compared to controls. After hatching, pre-exposed zebrafish larva showed signs of malformations (edema, bent and curving tail, necrosis) (Figure 2) and the malformation rate is significantly correlated with the microcystin contents of the extracts used (Pearson’s r = 0.979, P < 0.05). A reduction in total body length, measured at the beginning and in the end of hatching, was observed in all treatments. This reduction in body length was statistically significant in the 0.1 and 0.3 mg·L^−1^Eq MC-LR-treated groups. Nevertheless, effects on hatching rates and larval length at hatching are not significantly correlated with microcystin concentration (Pearson’s r is −0.729 and −0.809 respectively, P > 0.05).

**Figure 3.**
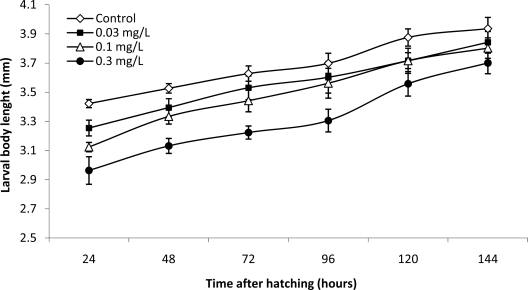
Effects of pre-exposure to a *Microcystis aeruginosa* natural bloom extract on the larval development of the zebrafish (*Danio rerio*). Values are mean ± S.E. of 30 animals per treatment.

The evolution of larval total body length presented in Figure 3 shows that there is a significant difference between control and pre-exposed groups during the first four days post-hatching. This difference becomes non-significant at the end of the test, moreover the specific growth rate of the larvae pre-exposed to the cyanobacterial extracts containing 0.1 and 0.3 mg·L^−1^ Eq MC-LR is significantly higher than that of the control larvae (Table 1).

**Figure 4.**
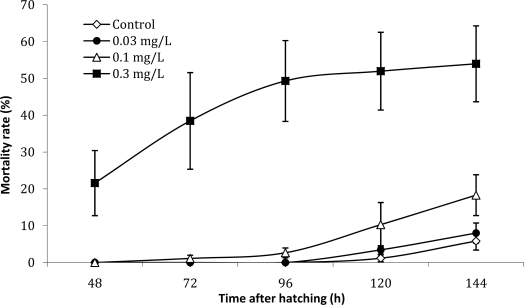
Mortality rate of the zebrafish (*Danio rerio*) larvae during six post-hatching days of pre-exposure to different treatment prepared from a *Microcystis aeruginosa* natural bloom extract.

During the larval stage, the fish pre-exposed to the highest used concentration (the extract containing 0.3 mg·L^−1^ Eq microcystin-LR) show a significant mortality rate (53% at the end of the test) which largely exceeds the data observed in the other treatments (Figure 4). Ninety six hours after zebrafish eggs hatching, the mortality rate starts increased gradually in the control and low concentrations, whereas it becomes more stable in larvae pre-exposed to the highest concentration.

One of the major objectives of this work was to provide detailed information about the effect of cyanobacterial blooms, with multiple microcystins variants, on embryolarval development of the zebrafish (*Danio rerio*). Although there has been a significant amount of research on toxicity of microcystins including the effects on aquatic organisms, the results of various experimental setups are often contradictory and several studies demonstrated microcystin-independent toxicities [11, 16, 27]. In our experiments, we approached the ecosystem situation when developing stages of aquatic organisms are exposed to cyanobacterial blooms with complex mixtures of organic molecules (not purified toxins). We studied the effects of a *Microcystis aeruginosa* bloom extracts on embryolarval development of the zebrafish (*Danio rerio*) at concentrations typical for naturally occurring cyanobacterial water blooms in Morocco [28, 29] as well as in many countries of the world. Cyanobacteria often accumulate in littoral zones and early-life stages of amphibians and fish [30] may be exposed to this biotic stress. Larval stages might be more affected than juveniles or adults due to their lower mobility and limited ability to avoid contamination [17].

In our experiments the highest toxicity (100% embryo mortality) was observed with the bloom extract containing 3 mg·L^−1^ Eq microcystin-LR (Table 1). This microcystin concentration is particularly high, but by using a broad range of microcystins concentrations we tried to determine the existence of a correlation between microcystins and the toxic effect observed, knowing that a cyanobacterial extract may contain other molecules that can affect the zebrafish embryos [24–26]. Besides lethal effects, cyanobacterial extracts also induced significantly malformations in surviving embryos (Figure 2). Our results indicate that cyanobacterial extracts significantly affect both viability and development of zebra fish embryos.

Our observations are in opposition with the study of Fischer and Dietrich [10] who observed no mortality, malformations or growth inhibitions in *Xenopus laevis* embryos exposed for 96 h to purified microcystins up to 2000 μg·L^−1^. Oberemm *et al*. [17] observed only a delay in the feeding rates of axolotl larvae (*Ambystoma mexicana*) exposed to 5 and 50 μg·L^−1^ of microcystin-LR and no mortality or morphological developmental changes were observed in axolotl as well as other amphibian species such as the smooth newt (*Triturus vulgaris*) and marsh frog (*Rana ridibunda*). Similarly, no developmental toxicity of microcystin-LR (up to 20,000 μg·L^−1^) was observed in experiments with the toad *Bufo arenarum* [31]. On the other hand the study of Best *et al*. [11] with brown trout alevins (*Salmo trutta*) showed significant effects of complex aqueous extracts of a *Microcystis sp*. strain on larval cardiac development but no changes were recorded with purified microcystin only.

Our observations on the role of microcystins in the overall toxicity to aquatic vertebrates are also supported by Dvorakova *et al*. [18] who observed developmental malformations in *X. laevis* embryos exposed to concentrations of purified microcystin-LR higher than 25 μg·L^−1^. More pronounced effects were observed after exposures to complex cyanobacterial extracts regardless of the microcystin content. As reported by Oberemm *et al*. [16, 17], exposure to crude cyanobacterial extracts containing microcystins had generally more pronounced effects on fish and amphibian embryos (malformations or mortalities), if compared to those obtained with pure toxins. Severe impairment of embryonic development has been observed in fish eggs after microinjection of microcystins [14, 23]. On the other hand, other studies with fish indicate that external exposure to microcystins might affect development of these aquatic vertebrates. For example, in the study with zebrafish (*Danio rerio*) Oberemm *et al*. [16] reported retarded larval growth already at 0.5 μg·L^−1^ of purified microcystin-LR and decreased survival was further observed at embryos exposed to 5 and 50 μg·L^−1^. Liu *et al*. [22] observed increase of developmental abnormalities and mortality together with hepatotoxicity and cardiotoxicity in embryos and larvae of loach *Misguruns mizolepis* exposed to dissolved MC-LR.

As described for many xenobiotics [20] the embryos may be more susceptible to cyanobacterial toxins than juvenile and adult fish. In fact in our work the exposure to the toxic extracts during the embryonic stage resulted in a significant increase in the mortality of eggs and a developmental delay (Table 1). The significant mortality rate in the larvae pre-exposed to the extract containing 0.3 mg·L^−1^ Eq MC-LR is probably a consequence of the large number of larvae with malformations inherited from the embryonic stage, which explains the high mortality of zebrafish larvae during the first three post-hatching days (Figure 4).

The existence of a significant difference in size between pre-exposed and control larvae (Figure 3), is influenced directly by the small size at hatching of the pre-exposed group (Table 1), and it is not related to a direct effect on zebrafish larvae. This is confirmed by the fact that the difference in size between pre-exposed and control larvae becomes not-significant at the end of the test (Figure 3).

The calculation of the specific growth rate shows that the growth of the larvae pre-exposed to the extracts containing 0.1 and 0.3 mg·L^−1^ Eq MC-LR is significantly higher than the growth of the control larvae (Table 1). This kind of growth carried out at rate higher than the normal is called “compensatory growth”. In the literature on fish growth, the term “compensatory growth” has been used to describe the accelerated growth by an individual after a period of growth depression [32]. Many organisms exhibit faster growth during recovery from stress than they do during normal conditions. The consequence is that animals experiencing a period of growth depression may achieve the same size-at-age as conspecifics experiencing environmental conditions that are more favorable [33]. Compensatory growth has been observed in a range of invertebrates and vertebrates [34, 35]. This kind of growth is identified by being significantly faster than the growth rate of control animals that have not experienced growth depression, held under comparable conditions. This accelerated growth eventually declines to growth rates typical of the control animals. Growth may be controlled by feedback mechanisms, which adjust growth rates to achieve a target trajectory [36, 37].

Thus normally to obtain a compensatory growth, two phases are needed: a first phase of stress during which the growth of the animals is reduced, followed immediately by the second phase during which the growth depressor disappear, what allows the resumption of the growth but at a rate below the physiological potential of the animal (compensatory growth). In our study the first phase corresponds to the exposed embryonic stage, and the second phase corresponds to the larval stage which begins immediately after zebrafish eggs hatching. The growth depressor is the toxic extract of *Microcystis aeruginosa* with microcystins, and the exposure is stopped during the larval phase. To our knowledge, this is the first time that a compensatory growth is observed in an animal after pre-exposure to a toxic cyanobacterial extract containing microcystins.

Most studies in fish caused compensatory growth following a period of total or partial food deprivation. The consequences of changing food quality on growth are also susceptible to compensation [38]. It has also been induced after a period of unseasonably low temperatures, which reduced the rate of food consumption in juvenile Atlantic salmon, *Salmo salar* [39, 40] or Atlantic cod, *Gadus morhua* [41] and during treatment of diseased rainbow trout, *Oncorhynchus mykiss* with hydrogen peroxide [42]. Spotted wolf-fish *Anarhichas minor,* displayed compensatory growth after being returned to normal oxygen conditions, following 75 days in hypoxic conditions [33]. In other studies, the origin of the reduced growth and the subsequent compensation has not been clear [43–46].

## 3. Experimental Section

### 3.1. Bloom sampling

**Figure 5.**
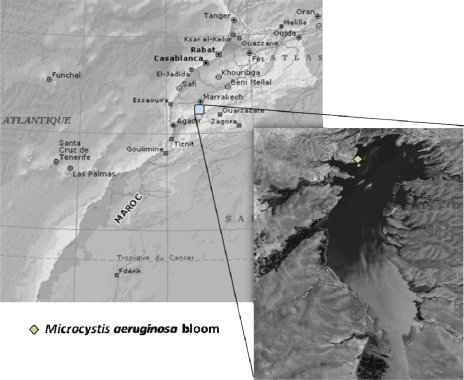
Localization of Lalla Takerkoust reservoir.

The cyanobacterial bloom material was collected from Lalla Takerkoust reservoir (Figure 5) with a 27 μm phytoplankton net in September 2005. The bloom-forming species was identified as *Microcystis aeruginosa* by both microscopy and PCR analysis. The collected samples were freeze-dried and stored until total microcystins quantification.

### 3.2. Microcystins detection and quantification

The toxin extraction and pre-purification were done according to Lawton *et al*. [49] using methanol, which was shown to be the most suitable solvent for microcystin extraction. Briefly, lyophilized cyanobacterial cells (250 mg) were extracted three times with 70% methanol (50–75 mg dry cells per mL of methanol). For each extraction, the suspension was centrifuged at 4,000×*g* (10 min, 4 °C). Afterwards, the supernatant was retained and the pellet was further extracted. The three methanol extracts were diluted with Milli-Q ultra-pure water to a final methanol concentration of 20% (v/v). For the microcystin pre-purification, the final extract was passed through Octadecyl silicagel ODS-C18 environmental Sep-Pak cartridges (1 g, Waters, Chromatography Division/Millipore Corp.). The ODS cartridges were previously activated with 100% methanol (40 mL) and Milli-Q ultra-pure water (40 mL). Then the diluted methanolic extract was applied to the cartridges that were washed with 20% methanol (20 mL) and ultrapure water (40 mL). The microcystins were finally eluted with 100% methanol (20 mL). The last collected fraction containing the toxins was completely evaporated at 40 °C and resuspended in methanol/Milli-Q ultra-pure water (1 mL, 50:50, v/v) and filtered through a GF/C glass filter before being analysed by HPLC.

Chromatographic analysis was performed on a Waters HPLC system (model 2695) equipped with a photodiode array detector (model 996). The column used was Chromolith C18 (250 mm×4.6 mm, 5 μm). The mobile phase system was: (A) H_2_O + 0.05% (v/v) trifluoroacetic acid (TFA), and (B) acetonitrile (MeCN) + 0.05% (v/v) TFA. During the HPLC running time of 55 min, the separation was achieved using a solvent gradient from 70% to 0% (A). The sample volume injected was 50 μL, and the mobile phase run at 1mL·min^−1^. The UV spectrum for each separated fraction was checked and the microcystins variants were preliminarily identified by their characteristic UV spectrum (max absorbance at 238 nm). Standard MC-LR, -YR and -RR were purchased from Calbiochem (Germany). MC-FR and -WR were purified in the Laboratory of Plant Physiology of the Autonomo s University of Madrid. Other microcystins different from the previous ones were quantified using microcystin-LR as a standard. The results are presented as MC-LR equivalent by adding the mass of all the variants found. MC(H4)-YR was identified by Liquid chromatography-mass spectrometry, the LC–MS experiments were carried out on an Agilent 1100 series HPLC system (Agilent Technologies, CA), consisting of a vacuum degasser, a binary pump, an autosampler and DAD detector, coupled to a hybrid quadrupole time of flight (QTOF) instrument (QStar/Pulsar i; Applied Biosystems, CA.) equipped with a turbospray ion source interface. The column used was Teknokroma, MED SEA18, and the mobile phase was a gradient of two eluents: (A) H_2_O + 0.1 % TFA, and (B) MeCN + 0.1 % TFA. Separation was achieved using the gradient B of Table 2. The chromatogram obtained in HPLC-MS, was identical to those obtained using HPLC-DAD.

Full scan MS spectra were acquired in the positive ion mode, using a source potential of 5000 V, over the mass range of 50–1,500 at 1 s. ESIMS/ collision induced dissociation (CID) mass spectra were measured using N_2_ as a collision gas (collision energy, 3 kV) in the pressure range of 65 Bar. The N_2_ drying temperature was set at 300 ºC, and the cone voltage was fixed at 70 V. Mass signals of unknown compounds with sufficient intensities (>1000 counts in accumulated spectra) were analyzed, and fragment patterns were compared with those from known or partly characterized MCs. All chemicals were of chromatographic grade (Scharlau Chimie Barcelona, Spain).

### 3.3. Preparation of the natural bloom extract

The cyanobacteria cell extract was prepared from the freeze-dried *Microcystis aeruginosa* bloom previously analyzed by HPLC-PDA (976 μg·g^−1^ Eq MC-LR). In order to release cell constituents, lyophilized cyanobacterial cells were extracted with 50% methanol and sonificated for 3 min in ice (Sonics Materials, Vibra Cell 50). After, the suspension was centrifuged at 23 000 rpm (10 min, 4°C), the supernatant was retained and filtered through a 0.2 μm sterile filter (Acrodisc, Polyethersulfone, VWR International) to exclude cell debris. At the beginning of the experiment, one stock of cell extract was prepared and kept at 4°C in the dark. Every 24h part of this stock was diluted to obtain four different concentrations (3; 0.3; 0.1 and 0.03 mg·L^−1^ Eq microcystin-LR) containing the same concentration of methanol (0.1%). In this study we used a natural cyanobacterial bloom extract without the purification process in order to mimic the natural conditions.

### 3.4. Exposure procedure

Zebra fish eggs were obtained from a broodstock maintained at the laboratory under the conditions described by Westerfield [50]. Fertilized eggs (3 hpf) were incubated in six-well cell culture plates (TC plates for suspension cells, sold by Sarstedt) in the dark at 25°C. Each well contained 5 mL of *Microcystis aeruginosa* natural bloom extract (4 dilutions with 3; 0.3; 0.1 and 0.03 mg·L^−1^ Eq microcystin-LR). For the control group, dechlorinated water is used as incubation medium. Every 24 hours the medium was renewed to ensure constant conditions during the 9 days of incubation. Oxygen and pH were monitored daily to ensure acceptable levels in the treatment and the controls (oxygen 5.6 mg·L^−1^ and pH 7–8 units). After hatching larvae were transferred to a medium without toxins (dechlorinated tap water) to follow the larval development under the new conditions. Each treatment and the control were done in six replicates (30 eggs per well, n = 30×6 = 180). Embryonic as well as larval development was observed throughout the entire test. Parameters monitored comprise hatching rate, hatching time, hatching duration, larval mortality rate and larval length. Aberrations of normal development as well as larval behavior were also recorded. Observation of embryonic and fish development was made under a Zeiss Stemi DV4 stereomicroscope. All photographs were taken by a Canon PowerShot A620 digital camera and analyzed using Adobe® Photoshop CS2. Larval standard length was measured from these images, using the UTHSCSA Image-Tool v3.00 program (developed at the University of Texas Health Science Center at San Antonio, TX, USA). The animals were handled in accordance with European Union regulations concerning the protection of experimental animals.

### 3.5. Calculations and statistics

$$\text{Specific}\;\text{growth}\;\text{rate}\;(\%)=(\frac{\text{final}\;\text{body}\;\text{lenght}-\text{initial}\;\text{body}\;\text{lenght}}{\text{time}})\times 100$$

Means, standard deviations, and standard errors for all experimental parameters were calculated using Microsoft® Excel 2003. One-way analysis of variance (ANOVA) and the Tukey’s test (SPSS 11.5) were carried out to determine whether treatments were significantly different from control group (*p < 0.05*). Pearson’s coefficient (*R* Pearson) was calculated to assess the existence of a correlation between microcystins contents of the bloom extracts used in this experiment and the parameters monitored.

## 4. Conclusions

Our results demonstrate significant embryo and larval toxicity of *Microcystis aeruginosa* natural bloom extracts in *Danio rerio* embryos. The effects were correlated with the amount of microcystins in the complex cyanobacterial sample. In this study, the embryo-toxicity of *Microcystis aeruginosa* bloom extracts is not related to the depletion of dissolved oxygen, because oxygen and pH were monitored daily to ensure acceptable levels in the treatment and the control (oxygen 5.6 mg·L^−1^ and pH 7–8 units). Our results showed the biotic stress related to the exposure of fish embryonic stages to toxic cyanobacterial blooms.

We found also that newly hatched zebra fish larvae seem to use compensatory growth to reduce the difference in size compared to the control fish. This is the first report of a compensatory growth process observed in an animal after a pre-exposure to a toxic cyanobacterial extract containing microcystins. Several factors could contribute to the compensatory growth observed after a period of growth depression. These include enhanced growth efficiency, reduced metabolic costs and reduced expenditure on locomotion.

## Figures and Tables

**Table 1.** Effect of different extracts obtained from *Microcystis aeruginosa* natural bloom on embryonic and larval development of the zebrafish (*Danio rerio*).

	Control	0.03 mg·L^−1^	0.1 mg·L^−1^	0.3 mg·L^−1^	3 mg·L^−1^
Time of beginning of hatching (h)	73±0	76±0	77±0	84±0	-
Hatching duration (h)	11±0	22±0	23±0	38±0	-
Hatching rate (%)	66.6±1.19^a^	52.2±1.82^b^	47.7±3^b^	45.5±3.44^b^	0
Malformations (%)	2.42±1.19^a^	7.11±0.73^a^	9.08±1.15^a^	18.05±2.44^b^	-
Larval length at hatching (mm)	3.15±0.04^a^	3.06±0.08^a^	2.84±0.08^b^	2.83±0.14^b^	-
Larval length after 90% of hatching (mm)	3.30±0.04^a^	3.25±0.05^a^	3.12±0.03^b^	3.13±0.05^b^	-
Specific growth rate (%)	0.24±0.01^a^	0.30±0.03^b^	0.40±0.02^c^	0.43±0.02^c^	-

Values are Mean ± Standard error.

Means in the same line sharing a common superscript are not statistically different (*p*≥0.05).

**Table 2.** Eluent gradient conditions used in LC-DAD. In all cases, eluent rate was set at 1 mL·min^−1^.

	Time (min)								
	0	3	33	40	50	55	60	62	65
Eluent A (%)	65	65	55	55	35	0	0	65	65
Eluent B (%)	35	35	45	45	65	100	100	35	35
